# Xanthogranulomatous Pyelonephritis in a Socioeconomically Disadvantaged Immigrant Child: A Case Report

**DOI:** 10.34172/aim.34417

**Published:** 2025-08-01

**Authors:** Çiğdem Arslan Alici, Şule Pektaş Leblebicier, Aykut Aykaç, Kazım Zararci, Yalçın Kara

**Affiliations:** ^1^Paediatric Urology Clinic, Health Sciences University Eskisehir City Hospital, Eskisehir, Turkey; ^2^Department of Paediatric Nephrology, Health Sciences University Eskisehir City Hospital, Eskisehir, Turkey; ^3^Department of Urology, Health Sciences University Eskisehir City Hospital, Eskisehir, Turkey; ^4^Department of Paediatric Intensive Care Unit, Health Sciences University Eskisehir City Hospital, Eskisehir, Turkey; ^5^Department of Paediatric Infectious Disease, Health Sciences University Eskisehir City Hospital, Eskisehir, Turkey

**Keywords:** Xanthogranulomatous pyelonephritis, Pediatric, Socioeconomic disadvantage, Immigrant, Staghorn stones

## Abstract

Xanthogranulomatous pyelonephritis (XGP) is a rare, chronic inflammatory kidney disease characterized by progressive destruction of the renal parenchyma, typically associated with obstructive uropathy and nephrolithiasis. It is especially uncommon in pediatric patients and may be mistaken for malignancy or other renal infections, leading to delays in diagnosis. We present the case of a 13-year-old socioeconomically disadvantaged and immigrant (SDI) girl who developed advanced XGP due to unmonitored renal stones and limited access to healthcare. She presented with prolonged fever, flank pain, weight loss, and fatigue. Imaging revealed a non-functioning, enlarged right kidney with multiple staghorn calculi. Despite initial antibiotic therapy and percutaneous nephrostomy drainage, her condition progressed, necessitating nephrectomy. Histopathological examination confirmed XGP with lipid-laden macrophages, granulomatous inflammation, and fibrosis. Stone analysis revealed a mixed composition, primarily calcium oxalate and apatite variants, consistent with chronic infection-related calculi. This case highlights the potential severity of XGP when diagnosis and management are delayed, particularly in SDI populations. Clinicians should consider XGP in children presenting with recurrent urinary tract infections, renal calculi, and nonspecific systemic symptoms. Early recognition and timely surgical intervention, guided by a multidisciplinary team, are essential to prevent irreversible renal damage and reduce morbidity in pediatric patients with complicated urinary tract conditions.

## Introduction

 Xanthogranulomatous pyelonephritis (XGP) is a rare and severe chronic inflammatory condition of the kidney, typically associated with chronic urinary tract obstruction. Although most commonly observed in middle-aged adults, it can occur in pediatric patients, where its presentation is even rarer. Factors such as recurrent urinary tract infections, nephrolithiasis, and socioeconomically disadvantaged and immigrant (SDI) status are significant risk factors contributing to delayed diagnosis and progression. Pediatric patients from low-income, frequently relocating households, including but not limited to families, may face challenges in continuity of care, increasing their vulnerability to advanced disease.

 Pediatric XGP is extremely rare, yet it accounts for approximately 16% of nephrectomies performed in children.^1^ Fewer than 300 pediatric cases have been documented in the literature to date,^2^ and the majority of these occur in children under 5 years of age.^3^ The disease is usually unilateral and often reported to involve the right kidney, although some studies suggest a predominance in the left kidney.^4^ These data underscore the rarity yet clinical significance of XGP in pediatric patients, highlighting the importance of early diagnosis and appropriate management to improve prognosis.

## Case Report

 A 13-year-old SDI girl presented with a two-month history of persistent right flank pain, intermittent fever, weight loss, and fatigue. Her medical history revealed a diagnosis of renal calculi at the age of 10, but she did not receive follow-up due to socioeconomic limitations and frequent relocations. On admission, her anthropometric parameters were below the 3rd percentile (height: 140 cm; weight: 32 kg). She appeared moderately ill, with pallor, tachycardia (HR: 120/min), hypotension (BP: 100/60 mm Hg), and a temperature of 38.1 °C. A palpable right-sided abdominal mass extending from the upper quadrant to the pelvis was detected. Laboratory results are summarized in Table 1.

**Table 1:** Laboratory Results in our Pediatric XGP Case

**Test**	**Result**
Hemoglobin (Hb)	6.1 g/dL
Hematocrit (Hct)	21.6%
CRP	98.9 mg/dL
Albumin	2.9 g/L
Phosphate	1.1 mg/dL
WBC (pre-op)	High (not quantified)
WBC (discharge)	5220/mm³
Hemoglobin (discharge)	13.6 g/dL
CRP (discharge)	7 mg/dL
Urine leukocytes	520
Urine erythrocytes	107
Urine culture	Escherichia coli (100,000 CFU/mL)
Nephrostomy culture	Proteus mirabilis

**Figure 1:**
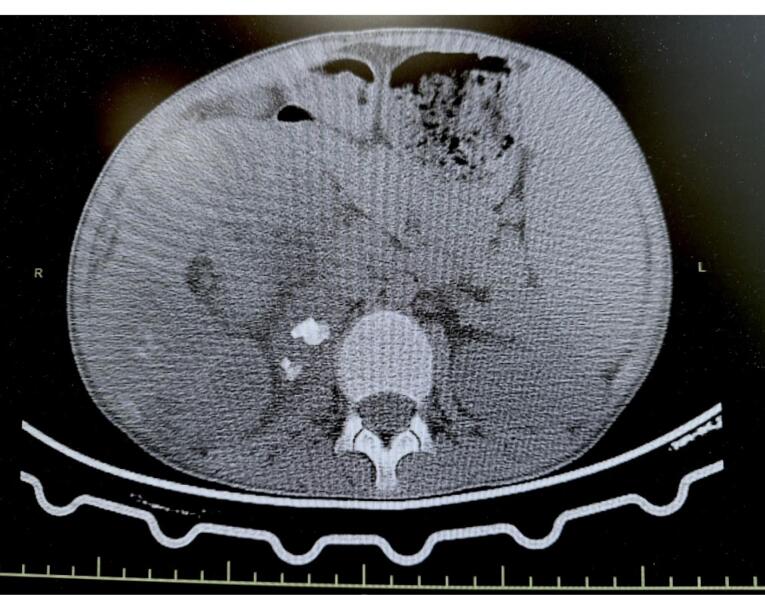
Bear Claw and Multiple Kidney Stones Seen on Abdominal CT

 Imaging revealed an enlarged, non-functioning right kidney with the characteristic ‘bear claw’ sign on contrast-enhanced computed tomography (CT) and multiple kidney stones measuring around 2 cm in size (Figure 1). 99mTc-dimercaptosuccinic acid (DMSA) nuclear scan showed that the affected kidney had no differential function. Family consent was obtained.

 Due to the patient’s septic state, percutaneous nephrostomy was performed, draining approximately 1400 mL of purulent material. Gram staining showed abundant polymorphonuclear leukocytes (PMNL) and gram-negative bacilli; culture of the material yielded *Proteus mirabilis*. Meropenem was administered, and the nephrostomy site was flushed with saline twice daily. Following drainage, meropenem treatment was initiated and continued for a total of three weeks. Despite this, a nephrectomy had to be planned. After the nephrectomy, four days of treatment were completed. The patient was discharged without antibiotic treatment and is being monitored every three months.

**Figure 2:**
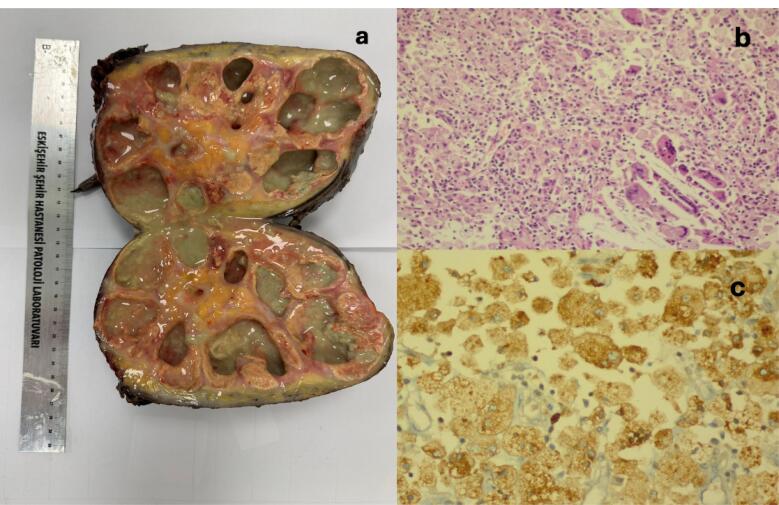
**a. **Appearance of a Kidney after Nephrectomy for Xanthogranulomatous Pyelonephritis, **b. **Xanthoma Cells, Cholesterol, and Crystal Clefts (H&E x 100), **c.** CD68 + Macrophages (Xanthoma Cells) (CD68 × 400)

 Gross pathological examination revealed a kidney measuring 18 × 11 × 10 cm, with severely dilated calyces containing grey-green, purulent fluid and three staghorn calculi (1–2.5 cm). Histopathological analysis confirmed XGP, revealing multiple yellow nodular areas 0.2–2 cm in diameter composed of foamy macrophages (CD68 + ), lymphocytes, and neutrophils (Figure 2). Stone composition analysis identified staghorn stones with weddellite (calcium oxalate), hydroxyapatite, fluoroapatite, and chlorapatite.

 Immune deficiency screening, serum immunoglobulin measurement, and lymphocyte subpopulation analysis (including lymphocyte subpopulation markers such as CD3, CD4, CD8, CD19, CD20, and CD56) were all within normal limits. QuantiFERON test and culture for tuberculosis were negative and a PCR test returned negative. Urinary calcium excretion was normal in both spot and 24-hour analyses; however, oxalate was elevated in the 24-hour urine. Genetic testing was undertaken. The patient was started on pyridoxine, and her dietary and fluid intake were regulated.

 The patient’s meropenem antibiotic therapy continued for 4 more days after the surgery. Postoperatively, the patient was monitored in the pediatric intensive care unit. Her condition improved significantly, and she was discharged on postoperative day 4. She was evaluated in the outpatient clinic control 10 days after discharge. Her general condition was good, her anemia (Hb: 14), electrolyte abnormalities and hypoalbuminemia (albumin: 4.1 gr/dL) were resolved.

## Discussion

 XGP is a rare, chronic inflammatory disease characterized by the progressive destruction of renal parenchyma, most commonly associated with obstructive uropathy.^5,6^ XGP diagnosis and management pose additional challenges in unmonitored and underserved patient populations. In the present case, progressive renal destruction developed in an SDI child as a consequence of inadequate follow-up for renal calculi, primarily due to socioeconomic barriers and frequent relocations.

 Given the rarity of XGP in pediatric patients, delays in diagnosis are not uncommon. Although the disease may affect individuals of all ages, it is most frequently diagnosed in middle-aged adults. The estimated global incidence of XGP is 1.4 cases per 100,000 individuals annually.^7^ Pediatric occurrences are exceedingly rare; since 1960, only 265 cases have been documented in the English-language medical literature,^8^ and just 12 cases have been reported in the Korean literature since the first description by Ahn et al in 1977.^9^

 A retrospective study by Stoica et al^10^ reviewed medical records of children diagnosed with XGP between 1963 and 2016. The mean age was 4.84 years (range: 1.1–14.81 years), with a male-to-female ratio of 1.35:1. All patients underwent nephrectomy. Common clinical features included systemic symptoms (62.1%), pain (60.6%), urinary tract infections (54.5%), and an abdominal mass (39.4%). Fever was present in 53% of cases. Laboratory abnormalities included anemia (86.3%), thrombocytosis (80.3%), and hypomagnesemia (65.1%). The current case shared many of these characteristics, including fever, pain, anemia, and hypomagnesemia.

 XGP represents an atypical and destructive variant of chronic pyelonephritis, in which the renal parenchyma is replaced by granulomatous tissue rich in lipid-laden macrophages.^11^ The pathological features of XGP were first described by Schlagenhaufer in 1916, and the term XGP was introduced by Putschar in 1934. Multiple etiologic mechanisms have been proposed, including urinary tract obstruction, chronic bacterial infection (especially with *P. mirabilis* and *Escherichia coli*) and metabolic disturbances, although the exact pathogenesis remains poorly understood.^12-14^

 In this case, limited health literacy and language barriers contributed significantly to delayed healthcare access and disrupted continuity of follow-up, underscoring the importance of culturally sensitive healthcare delivery systems.

 XGP typically affects a single kidney and presents with nonspecific clinical features such as abdominal pain, flank mass, unexplained fever, weight loss, pyuria, and, less commonly, hematuria.^15-17^ Laboratory findings are often non-specific and may include mild anemia, leukocytosis, and elevated C-reactive protein levels.^18,19^ The patient in this report exhibited similar findings, with leukocytosis and anemia noted on admission.

 Stone analysis and cultures revealed *E. coli* in the urine and *P. mirabilis* in the nephrostomy drainage, both of which are commonly associated with XGP-related infections. These findings align with previously reported microbial profiles in XGP patients.^20^

 XGP is categorized into three stages based on the extent of inflammation: Stage 1, confined to the renal parenchyma; Stage 2, involving the perirenal adipose tissue; and Stage 3, characterized by invasion into adjacent structures such as the retroperitoneum, diaphragm, or psoas muscle.^15^ Radiological and clinical findings in this patient were consistent with Stage 2–3 disease.

 The patient’s anemia was likely secondary to chronic inflammation and resolved following nephrectomy, consistent with the natural course of inflammation-related anemia.^21^

 Nevertheless, inadequate management of renal calculi and recurrent urinary tract infections due to limited access to healthcare services in SDI patients may play a critical role in the development of XGP.

 Although imaging modalities such as ultrasound can identify XGPN, contrast-enhanced CT and magnetic resonance imaging (MRI) offer high sensitivity and can detect swollen non-functional kidneys, along with invasion into nearby tissues.^22,23^ In the present case, abdominal contrast-enhanced CT findings revealed a globally destroyed right kidney with numerous large renal stones, a classic characteristic that is documented in approximately 80% of XGPN cases.^2^ The staghorn calculi in this case were composed primarily of calcium oxalate dihydrate and apatite variants, consistent with stones associated with chronic urinary tract infection.^20^

 The precise standard for XGPN is pathological diagnosis, and the pathological features in question include the foam cells formed by substantial macrophage infiltration inside the renal parenchyma and the multiple granulomas composed of various inflammatory components (e.g. neutral granulocytes, lymphocytes, plasma cells, cholesterol clefts and multinucleated giant cells). The foam cells are specific diagnostic indicators.^24^ Our case showed the same pathological features. The low incidence of XGPN, coupled with its lack of specificity in clinical and imaging manifestations, results in a low diagnostic rate.^24^

 The management of XGPN varies in the literature according to the extent of the disease. In diffuse forms, nephrectomy is usually the standard treatment. In focal forms, partial nephrectomy, drainage of the perirenal/kidney abscess and concomitant antibiotic therapy have been recommended, but since focal XGPN resembles renal tumors, most cases proceed to renal exploration.^25,26^ As there is currently no clear guideline for the management of XGPN, conservative and surgical treatments should be evaluated individually for each case. In one case in the literature, a percutaneous nephrostomy tube was placed in an attempt to salvage the kidney, but conservative treatment eventually failed.^18^ Our review of the literature suggests that medical treatment works in some cases of focal XPN, but has not been tested adequately in diffuse XPN. In the present case, percutaneous nephrostomy tube insertion was elected, but nephrectomy was performed due to the absence of differentiated renal function and elevated acute phase reactants.

 This case highlights the challenges faced in the diagnosis and management of pediatric XGP, particularly in SDI populations. Whilst the association between pediatric XGP and staghorn stones has been previously described, our findings underscore the critical role of social determinants of health (such as limited access to healthcare, inadequate follow-up, and frequent relocation) in the progression of this rare condition. The resulting diagnostic delays and advanced disease stages observed in vulnerable children reflect broader health disparities. Clinicians should maintain a high index of suspicion for XGP in children with chronic urinary symptoms and a history of urolithiasis, especially when consistent care is hindered by socioeconomic constraints. Early imaging, routine follow-up, and multidisciplinary management are essential to reduce morbidity and preserve renal function in this population. This case adds to the limited pediatric XGP literature by illustrating how socioeconomic instability and care discontinuity (rather than stone burden alone) can drive disease progression.

## Conclusion

 This case demonstrates the challenges associated with the diagnosis and management of XGP in paediatric and immigrant patients. Failure to detect and treat recurrent urinary tract infections and renal calculi in a timely manner can result in irreversible kidney damage. Given that socioeconomic barriers represent a significant impediment to migrant patients accessing health services, health policies should be developed to ensure the follow-up of these patients. Early diagnosis and a multidisciplinary approach can reduce morbidity and mortality due to XGP.
